# Anthropogenic sources of underwater sound can modify how sediment-dwelling invertebrates mediate ecosystem properties

**DOI:** 10.1038/srep20540

**Published:** 2016-02-05

**Authors:** Martin Solan, Chris Hauton, Jasmin A. Godbold, Christina L. Wood, Timothy G. Leighton, Paul White

**Affiliations:** 1 Ocean and Earth Science, National Oceanography Centre, Southampton, University of Southampton, Waterfront Campus, European Way, Southampton, SO14 3ZH; 2 Centre for Biological Sciences, Faculty of Natural and Environmental Sciences, University of Southampton, Highfield Campus, Southampton, SO17 1BJ; 3 Institute of Sound & Vibration Research, Faculty of Engineering and the Environment, University of Southampton, Southampton, SO17 1BJ.

## Abstract

Coastal and shelf environments support high levels of biodiversity that are vital in mediating ecosystem processes, but they are also subject to noise associated with mounting levels of offshore human activity. This has the potential to alter the way in which species interact with their environment, compromising the mediation of important ecosystem properties. Here, we show that exposure to underwater broadband sound fields that resemble offshore shipping and construction activity can alter sediment-dwelling invertebrate contributions to fluid and particle transport - key processes in mediating benthic nutrient cycling. Despite high levels of intra-specific variability in physiological response, we find that changes in the behaviour of some functionally important species can be dependent on the class of broadband sound (continuous or impulsive). Our study provides evidence that exposing coastal environments to anthropogenic sound fields is likely to have much wider ecosystem consequences than are presently acknowledged.

Anthropogenic sound associated with human activity is likely to disproportionately affect coastal and marginal shelf sea habitats because ~40% of the world’s population live within 100km of the coast at a density of more than 3 times the global average^1^,^2^. Although underwater sound is generated by many natural sources, including surface wave action, weather, glacier calving and animal communication, the extent to which sound sources associated with human activity contribute to ambient sound budgets has doubled every decade for the last 6 decades^3^,^4^. Yet, major gaps in understanding remain about whether man-made sounds affect species activities in relation to the functioning of ecological systems^5^,^6^. It is known that sources of continuous (characteristic of areas close to shipping lanes, commercial harbour environments and dredging activities) and impulsive (characteristic of the piling associated with marine civil engineering, construction and infrastructure projects) broadband sound can cause mortality^7^,^8^ in marine fauna, but exposure to such sounds are more likely to cause acute physiological effects^9^,^10^ or can alter the sound signature of specific locations^11^,^12^, thereby affecting the way species interact with one another^13^,^14^,^15^ and/or their environment^16^,^17^,^18^. As sound can propagate over great distances, it is reasonable to suggest that a large proportion of a species’ distributional range^19^,^20^,^21^ will be exposed to anthropogenic sound fields and, considering the temporal persistence of major shipping routes and typical offshore installations, such exposure may extend across multiple generations^8^,^22^,^23^.

Although acute responses to intense underwater sounds, including those created during the operation of seismic arrays, have generated considerable interest, the more significant risk to populations and ecosystems is likely to stem from the less visible effects of chronic exposure. Some species (fish, marine mammals) avoid, or minimise, exposure to sound through temporary population displacement^24^,^25^; however, the majority of coastal and shelf invertebrate species are sedentary and are unable to evade the local acoustic environment. Importantly, the persistence of species in a noisy environment does not equate to a null response; the effects of sound may not be lethal, but could have significant functional, fitness and ecological consequences that cannot be detected by survey alone. Previous work on sediment-dwelling invertebrates has demonstrated that a variety of changes in the abiotic environment [e.g. flow^26^, habitat configuration^27^, environmental regime variation^28^, ocean acidification and warming^29^] can affect species’ behaviour and, subsequently, important ecosystem properties such as nutrient turnover and primary production. Elsewhere, studies have shown that increased sound exposure can reduce relative individual fitness and affect community structure^8^,^22^,^30^,^31^. It is also known that context-dependent changes to organism physiology can alter species’ behaviour that pre-empt measureable changes in a species’ functional contribution to ecosystem properties^32^,^33^. This presents the possibility that increased exposure to different types of intense underwater sound may adversely impact the faunal mediation of important ecosystem processes that underpin the delivery of benefits to society, including carbon storage and nutrient cycling. If so, we will need to better understand when species are at risk from sound exposure and how this will compromise valued ecological functions^34^.

Here, we establish whether exposure to Continuous Broadband Noise (CBN) and Impulsive Broadband Noise (IBN) affects the physiology and behaviour of three representative and functionally important benthic invertebrate species (the clam, *Ruditapes philippinarum*; the decapod, *Nephrops norvegicus*; and, the brittlestar, *Amphiura filiformis*). As most physiological^10^,^35^ and behavioural^36^ observations of the acute responses of invertebrates to sound fields have only considered short-term (minutes) single and repeat exposures to CBN that likely reflect temporally ephemeral shock responses, or subsequent habituation^35^, we specifically assess time integrated faunal responses to different classes of sound field typically encountered in areas of offshore industrial activity. Our *a priori* expectation was that alternative sound fields may affect species’ behaviour in a number of ways, including changes in vertical positioning within the sediment profile and periods of valve closure in bivalves or altered burrowing activity in crustaceans and ophiuroid brittlestars, and that any observed responses would differ between species and/or with exposure to different sound classes^37^. Further, we considered that the underlying mechanism behind these changes in behavior would be a function of perturbations in the delivery of oxygen to active tissues within individuals, affecting the balance of aerobic and anaerobic metabolism and subsequent tissue biochemistry^38^. In turn, we speculated that these responses would affect particle-mixing and fluid transport behaviour, important mediators of nutrient regeneration in benthic environments.

## Results

### Effects of sound fields on organism tissue biochemistry

We found no evidence that exposure to continuous or impulsive sound fields over 7 days affected tissue concentrations of glucose (*Ruditapes philippinarum*, L-ratio = 0.393, d.f. = 2, p = 0.822; *Nephrops norvegicus*, L-ratio = 4.439, d.f. = 2, p = 0.109; *Amphiura filiformis*, L-ratio = 2.967, d.f. = 2, p = 0.227; Supplementary Figure S1) or lactate (*R. philippinarum*, F = 3.378, d.f. = 2, p = 0.068, Supplementary Model S1; *N. norvegicus*, L-ratio = 2.829, d.f. = 2, p = 0.243; *A. filiformis*, L-ratio = 0.389, d.f. = 2, p = 0.824; Supplementary Figure S2). Pearson product moment correlations between lactate and glucose showed that changes in glucose mobilisation (tissue glucose concentrations) were not associated with changes in anaerobic metabolism (*R. philippinarum*, r = 0.24, t = 0.887, d.f. = 13, p = 0.39; *N. norvegicus*, r = 0.28, t = 1.053, d.f. = 13, p = 0.31; *A. filiformis*, r= 0.39, t = 1.521, d.f. = 13, p = 0.15). None of the species investigated here showed any significant changes in glycolytic activity, as evidenced from changes in tissue glucose concentrations, or any accumulation of tissue lactate that could be associated with specific sound field treatments, although intra-specific responses were highly variable (Supplementary Table S1) and may have masked these effects.

### Effects of sound fields on species’ behaviour and ecosystem process

**Figure 1:**
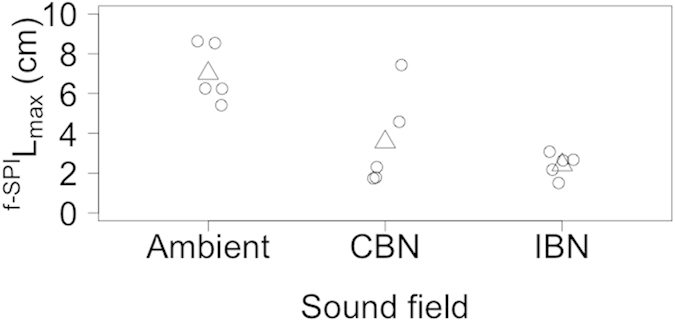
The effect of sound field on the maximum mixed depth (^f-SPI^L_max_, cm) of sediment particles for *Nephrops norvegicus*. Data points (open circles) have been horizontally jittered for clarity. Model predictions (open triangles) from the minimal adequate linear regression model (Supplementary Model S2) with GLS estimation (incorporating sound field as a variance covariate) are indicated. Positive values indicate increased particle reworking activity. Sound fields: Ambient, Gaussian sound spectrally shaped to mimic background sound; CBN, ambient + continuous broadband sound (Supplementary Sound 1); IBN, ambient + impulsive broadband sound (Supplementary Sound 2).

The maximum depth of sediment particle redistribution (^f-SPI^L_max_) for *R. philippinarum*, (ambient sound field, 2.25–4.52 cm; CBN sound field, 3.58–5.52 cm; IBN sound field, 2.27–4.93 cm) and *A. filiformis* (ambient sound field, 5.06–7.20 cm; CBN sound field, 5.36–7.13 cm; IBN sound field, 5.25–7.97 cm) was unaffected by sound field (L-ratio = 1.718, d.f. = 2, p = 0.424, and L-ratio = 0.374, d.f. = 2, p = 0.829 respectively; Supplementary Figure S3). For *N. norvegicus*, the mean (±95% CI) ^f-SPI^L_max_ under an ambient sound field (7.014 ± 1.827 cm) was reduced (L-ratio = 13.911, d.f. = 2, p = 0.001; Fig. 1, Supplementary Model S2) in the presence of CBN (coefficient = −3.448 ± 1.280 s.e., t = −2.693, p = 0.0196) and IBN (coefficient = −4.602 ± 0.711 s.e., t = −6.475, p < 0.0001). These changes reflected a response to the presence of a novel sound field as there was no difference in maximum mixing depth between CBN and IBN sound fields (coefficient = 1.154 ± 1.131 s.e., t = 1.021, p = 0.328). The mean (^f-SPI^L_mean_, Supplementary Figure S4) and median (^f-SPI^L_med_, Supplementary Figure S5) depth of sediment mixing, whether mediated by *R. philippinarum* (^f-SPI^L_mean_, L-ratio = 0.113, d.f. = 2, p = 0.945; ^f-SPI^L_med_, L-ratio = 2.975, d.f. = 2, p = 0.226)*, N. norvegicus* (^f-SPI^L_mean_, L-ratio = 2.883, d.f. = 2, p = 0.237; ^f-SPI^L_med_, L-ratio = 1.920, d.f. = 2, p = 0.383) or *A. filiformis* (^f-SPI^L_mean_, L-ratio = 3.362, d.f. = 2, p = 0.186; ^f-SPI^L_med_, F = 1.617, d.f. = 2, p = 0.239), were not affected by sound field.

**Figure 2:**
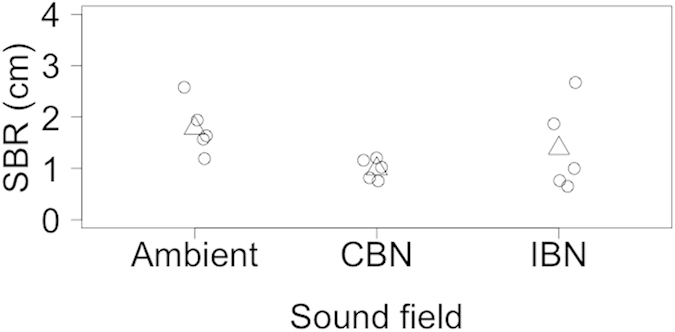
The effect of different sound fields on surface boundary roughness (SBR, cm) for *Ruditapes philippinarum*. Data points (open circles) have been horizontally jittered for clarity. Model predictions (open triangles) from the minimal adequate linear regression model (Supplementary Model 3) with GLS estimation (incorporating sound field as a variance covariate) are indicated. Positive values indicate increased activity within the sediment-water interface. Sound fields: Ambient, Gaussian sound spectrally shaped to mimic background sound; CBN, ambient + continuous broadband sound (Supplementary Sound 1); IBN, ambient + impulsive broadband sound (Supplementary Sound 2).

Whilst there was no affect of sound field on the surficial sediment reworking activities (Surface boundary roughness, SBR) of *N. norvegicus* (L-ratio = 2.193, d.f. = 2, p = 0.334; Supplementary Figure 6a) and *A. filiformis* (L-ratio = 0.033, d.f. = 2, p = 0.983; Supplementary Figure 6b), our analyses reveal that *R. philippinarum* decreases surficial activity (reducing surface boundary roughness) with exposure to broadband sound fields (L-ratio = 7.646, d.f. = 2, p = 0.022, Fig. 2). Closer examination of the minimal adequate model coefficients (Supplementary Model S3) reveals that the mean (±95% CI) surface boundary roughness under an ambient sound field (1.784 ± 0.644 cm) was reduced in the presence of CBN (coefficient = −0.792, t = −3.182 ± 0.249 s.e., p = 0.008), but not in the presence of the IBN (coefficient = −0.393 ± 0.450 s.e., t = −0.874, p = 0.399) sound field (Fig. 2). It is tempting to speculate that the increases in standard deviation (σ) within this treatment (σ_IBN_ = 0.861, compared to σ_ambient_ = 0.519 and σ_CBN_ = 0.200) may reflect intra-specific differences in response to sound field exposure (Supplementary Table S1).

**Figure 3:**
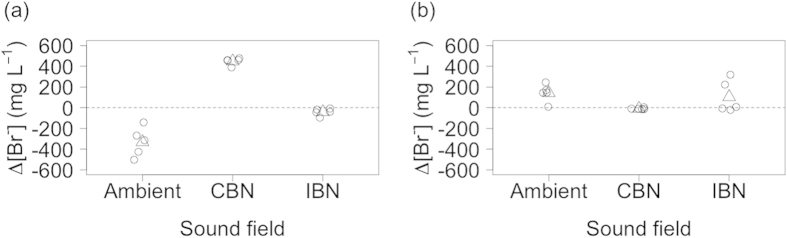
The effect of different sound fields on bioirrigation activity (∆[Br^−^], mg L^−1^) for (**a**) *Ruditapes philippinarum* and (**b**) *Nephrops norvegicus*. Data points (open circles) have been horizontally jittered for clarity. Model predictions (open triangles) from the minimal adequate linear regression models (Supplementary Models 4 and 5) with GLS estimation (incorporating sound field as a variance covariate) are indicated. Negative values indicate increased bioirrigation activity. Sound fields: Ambient, Gaussian sound spectrally shaped to mimic background sound; CBN, ambient + continuous broadband sound (Supplementary Sound 1); IBN, ambient + impulsive broadband sound (Supplementary Sound 2).

Analysis of the change in bromide concentrations over 4 h (∆[Br^−^], Fig. 3) revealed consistent bioirrigation activity (mean ± 95% CI, mg L^−1^), irrespective of sound field for *A. filiformis* (ambient, 45.231 ± 102.657; CBN, 28.286 ± 58.884; IBN, −5.500 ± 38.587; overall, 22.673 ± 32.043; L-ratio = 3.217, d.f. = 2, p = 0.200; Supplementary Figure S7), but not for *R. philippinarum* (L-ratio = 36.951, d.f. = 2, p < 0.0001; Supplementary Model S4) or *N. norvegicus* (L-ratio = 10.509, d.f. = 2, p = 0.0052; Supplementary Model S5). There was a much more marked bioirrigation response from *R. philippinarum*. Indeed, examination of the minimal adequate model coefficients for *R. philippinarum* (Supplementary Model S4) reveals that most bioirrigation activity (mean ± 95%CI, mg L^−1^) occurs under an ambient sound field (−330.607 ± 173.618), an intermediate level of bioirrigation activity occurs under an IBN sound field (−41.025 ± 42.501), and least bioirrigation activity occurs under a CBN sound field (448.497 ± 42.654). For *N. norvegicus* (Supplementary Model S5), bioirrigation activity (mean ± 95%CI, mg L^−1^) increases under CBN (−8.458 ± 9.594) relative to an ambient sound field (142.771 ± 105.348; coefficient = −151.22820 ± 38.100 s.e., t = −3.969, p = 0.002), but bioirrigation activity under CBN is no different to that observed under the IBN condition (104.152 ± 193.671; coefficient = 112.610 ± 69.840 s.e., t = 1.612, p = 0.133). For all three species, there was little to suggest that bioirrigation performance was more variable under sound exposure (Supplementary Table S1).

## Discussion

We have demonstrated, for invertebrate species that do not rely on acoustics for communication, that exposure to fully constrained sources of sound can result in behavioural responses that alter how species mediate ecosystem processes known to be key determinants of functioning. Importantly, however, differences in sound field characteristics can elicit different response patterns^37^ that appear to be proportional to the type of anthropogenic sound field that is encountered. In the case of *Nephrops norvegicus*, we show that the addition of either anthropogenic sound source repressed burying and bioirrigation behaviour and we observed considerably reduced locomotion activity. For *Ruditapes philippinarum*, the introduction of an anthropogenic sound source elicited a typical stress response where individuals reduce surface relocation activity, move to a position above the sediment-water interface, and close their valves. These responses reduce the capacity of the organism to mix the upper sediment profile and prevent suspension feeding from taking place. Whilst the behavioural responses observed in *R. philippinarum* and *N. norvegicus* provide direct evidence for the alteration of species-environment relations that will affect ecosystem properties, we were not able to statistically verify similar changes in behaviour for *Amphiura filiformis.* This matters because a finding such as this could be taken as evidence that a species is unaffected by exposure to anthropogenic sound. However, in this instance, closer examination of the data reveals that exposure to sound compromised physiological processes in a number of individuals (indicated by increased variability in response) that, in turn, corresponds to increased variability in some, but not all, aspects of bioturbation behaviour. Furthermore, for some species, our findings suggest that there is greater scope to acclimatise to one type of sound field over that of another because intraspecific differences in response mean that at least a subset of the population is capable of physiological and/or behavioural adjustment. These sources of response variability can lead to a lack of statistically significant patterns, but does not exclude the possibility that responses to environmental sound can be subtle and may take extended periods of time to be expressed across a population or become detectable at an ecosystem level. Indeed, a consistent feature in all of the response variables within this study, irrespective of species identity, is that responses associated with sound exposure required a specific model fitting approach to incorporate this source of unequal variance. This requirement within our analysis implies that the extent of species’ response is not solely dependent on exposure to sound. Instead, the response to sound exposure is likely moderated by a variety of attributes that are expressed at the level of an individual, including exposure history, environmental context and physiological condition. Importantly, this means that species responses to sound exposure will not necessarily manifest themselves consistently across all elements of a physiological process, behaviour or ecological property within the same timeframe or across locations that share similar environmental settings.

An important aspect of our study was the identification of null responses. None of the species investigated here showed any significant changes in glycolytic activity, as evidenced from changes in tissue glucose concentrations, or any accumulation of tissue lactate that could be associated with specific sound field treatments. In the case of lactate accumulation, the most notable response was that of *R. philippinarum* when exposed to an IBN sound field. Interestingly, two individuals within this treatment failed to accumulate lactate to a detectable level; however, the remaining three individuals accumulated much higher quantities of tissue lactate within the adductor muscle because they had closed their valves for an extended period of time, a known avoidance behaviour that can require the individual to respire anaerobically. This observation is not trivial, because lactate is not preferentially accumulated in bivalves as it leads to tissue acidosis and, potentially, shell dissolution or etching that weakens the valve over time. In general, bivalves accumulate succinate or opine molecules first, as end products of anaerobic metabolism^39^,^40^. Succinate, or succinic acid, is a weaker acid than lactic acid and does not present the same magnitude of tissue acidosis as it is accumulated^41^. Hence, the observations made here indicate that the pathways of succinate or opine accumulation have been exhausted following exposure to anthropogenic sound fields and at least some individuals had started to accumulate lactate at levels likely to cause harm if sound exposure continued over prolonged periods. If such delay in the expression of physiological responses following sound exposure is widespread, these observations caution against over-emphasizing the results of short-term studies^29^ and raise important questions about the level of certainty associated with current perspectives of the ecological consequences of sound exposure.

Scaling the individual impacts of marine sound to community and system level effects is key to the prediction and monitoring of future impacts within coastal and marginal shelf seas, yet our findings indicate that this may be challenging for some species and processes. It is important to emphasize, however, that the intra- and inter-specific variability in response documented here also provides an opportunity; the ecosystem consequences of anthropogenic sound fields are unlikely to be linearly related to exposure, providing an opportunity to minimise or mitigate the ecological impact of sound at identified thresholds, timings and/or locations. The determination of when and how physiological responses to sound exposure translate into ecologically relevant changes in behaviour, however, will not be straightforward, especially where multiple sources of disturbance are co-located^42^,^43^ or sound fields overlap^44^. Field studies of fish behaviour in the vicinity of offshore wind farms have reported complex interactions in demersal fish where aversion behaviour is partially offset by an attraction to emplaced structures^45^, whilst others have reported that acoustic disturbance can directly affect the likelihood of survival by increasing aggressive interactions^46^ in mammals or compromising foraging and anti-predator behaviour^36^,^47^ in invertebrates and fish. It is also known that thresholds of sound that elicit such changes in behaviour will vary, not least because species have a capacity for habituation^48^ but also because they may respond differently depending on the timing (e.g. in relation to cyclic periodicity^49^ and life phase^23^, soundscape complexity^50^ or, as shown here, the class^37^ of the sound field). This matters because the structural properties of the community are likely to be retained following periods of sound exposure, but the functional role and way in which species interact with their environment and with one another may well change^29^,^51^, temporarily or permanently, following the onset of exposure to anthropogenic sound.

Our findings lend support to the growing realization that the ability to regulate, legislate, monitor and predict the effect of anthropogenic sound fields on marine life is severely hampered by a dearth in knowledge^5^,^6^,^52^,^53^. Furthermore, a disproportionate focus on impact-related responses in the literature means that the reporting of an insignificant outcome is rare^54^ such that the extent and relevance of negative or neutral responses, including the potential for species recovery^55^, is largely unknown^56^. Whilst the impacts of anthropogenic sound fields on ecosystem services have been demonstrated at larger scales in terrestrial systems^57^, they have not been addressed in marine systems; this study provides an early precedent for considering that sound may directly and indirectly alter how species mediate ecosystem functioning. Rapid improvements in our understanding of the impacts of sound fields, particularly in marine mammals^58^ and fish^53^, but also in squid^59^, have been instructive, but have the potential to bias regulatory strategy by over-emphasizing information acquired from a limited number of short-term response variables achieved across a small subset of higher trophic level species. Although this limitation has been recognized for some vertebrate groups^60^, consideration of ecologically- and commercially-important benthic invertebrate groups is almost non-existent. Yet, in many cases, it is these lower trophic groups that play a dominant role in mediating many essential ecosystem processes. Importantly, as the results of this study stress, the effects of anthropogenic sound fields on functionally important species in lower trophic levels have the potential to be substantive, such that their exclusion from impact assessments is likely to lead to an under-appreciation of the effects of anthropogenic sound in offshore environments. Whilst this view argues for the achievement a more reliable assessment of the role of sound in shaping biodiversity-functioning relations, a principal challenge going forward will be to prioritize the ecological risks of sound exposure for key communities and habitats, whilst maintaining and building a balanced mechanistic understanding of why and when species are at risk, and whether this matters for a range of ecosystem properties over the long-term^61^.

## Methods

### Invertebrate fauna and sediment collection

Specimens of the Manila clam *Ruditapes philippinarum* were supplied by Othniel Shellfish of Poole, Dorset and acclimated to aquarium conditions for two weeks. During the acclimation period clams were fed to excess on alternate days with a mixed algal diet consisting of the species *Isochrysis galbana, Tetraselmis suecica, Pavlova lutheri, Phaeodactylum tricornutum* and *Chaetoceros ceratosporum*. Creel-caught decapod crustaceans *Nephrops norvegicus* sourced from Scotland were supplied from Portland Shellfish, Weymouth and acclimated to aquarium conditions for two weeks during which time they were held communally and fed on alternate days with a mixed bivalve diet of *Ruditapes philippinarum, Crepidula fornicata* (slipper limpets) and *Mytilus edulis* (mussels). Ophiuroid brittlestars *Amphiura filiformis* were collected from Loch Linnhe (west coast of Scotland) using a Van Veen grab deployed from RV *Seol Mara* (Scottish Marine Institute, Oban, Scotland). Individuals of *A. filiformis* were transported to Southampton in aerated water baths and acclimated to aquarium conditions for a one week. Sediment for all species treatments was collected from the Solent Estuary (Southampton) or Loch Linnhe (for *A. filiformis*), sieved (500 μm mesh) in a seawater bath to remove macrofauna, allowed to settle for 24 h to retain the fine fraction (less than 63 μm) and homogenized. Species were fed on alternate days throughout the experiment (*R. philippinarum*, 200 ml of *Pavlova lutheri* at a density of 4 × 10^6^ cells ml^−1^ to give a final density of approximately 1400 cells ml^−1^; *N. norvegicus*, 1.5 g_ww_ of *C. fornicata*; *A. filiformis*, 0.02 g of Aquarian® Tropical Fish Flakes). Visual inspections (5 times day^−1^) were used to identify any changes in species behaviour.

### Experimental design

We assembled replicate monocultures of each macrofaunal species (*N. norvegicus, A. filiformis* and *R. philippinarum*) at representative natural densities (1, 10 and 2 individuals aquarium^−1^, equivalent to ~4 ind. m^−2^, ~700 ind. m^−2^ and 240 ind. m^−2^, respectively) in transparent perspex cube aquaria (L × W × H: *N. norvegicus*, 45 × 45 × 45 cm; *A. filformis* and *R. philippinarum* 12 × 12 × 30 cm) containing sediment to a depth of 10 cm (15 cm in *N. norvegicus*) and seawater (15 °C, salinity 33, 10 μm filtered and UV sterilised). Aquaria were continually aerated and maintained in the dark. Short-term exposure (7 days) to each of three sound fields (ambient sound, ambient + CBN and ambient + IBN) was investigated for each of our measures of organism physiology (metabolic processes) and ecosystem process (bioturbation, bioirrigation) in an acoustically shielded circular seawater reservoir (see below). Aquaria were arranged in a random order 1m from the central sound source. Each Species_[n=3]_ × Sound field_[n=3]_ treatment was replicated 5 times, requiring a total of 45 aquaria.

### Generation of sound fields

Exposures to sound fields were conducted in a circular (2.4 m diameter, 0.75 m deep) seawater-filled (to 32 cm) fibreglass reservoir with anti-vibration shielding; severe acoustical impedance mismatch at the tank walls and the water-air interface causes over 99% of the acoustical energy to reflect, so the main conduit for unwanted sound transmission into the reservoir is through the base (here stopped by standard vibration isolation supports). As natural ecosystems are not silent, we generated a background (ambient) sound field typical of offshore shelf environments that are unaffected by acute sources of anthropogenic sound^62^. The ambient acoustic fields in the tank consisted of Gaussian sound spectrally shaped to mimic naturally occurring background sound fields across as wide a bandwidth as can be achieved. Three different playback conditions were considered (ambient, ambient + continuous broadband sound [CBN], ambient + impulsive broadband sound [IBN]). In each instance the sound spectrum of the source signal was shaped so that, in the frequency band 100 Hz–2 kHz, the spectrum of the sound recorded at the monitor hydrophone matched the input spectrum. In the quiet condition Gaussian white sound was replayed at a level to mimic oceanic ambient sound in sea-state 3–4. For the continuous sound condition, a recording (1 min duration, continuously looped) of a ship made in the English Channel at a distance of ~100 m was used (Supplementary Sound 1, Supplementary Figure S8). Similarly, for the impulsive sound condition, we used a recording (2 min duration, continuously looped) of a wind farm mono-pile being driven in the North Sea; the original recording was made at a distance of ~60 m distance (Supplementary Sound 2, Supplementary Figure S9). When replayed in the tank the sound pressure levels at the monitor hydrophones for the continuous sound case were typically in the region of 135–140 dB re 1 μPa, whereas for the impulsive sound, the sound exposure levels (SEL) were approximately 150 dB re 1 μPa^2^ s.

A pair of Electro-Voice UW-30 underwater speaks were positioned in the centre of the seawater reservoir, oriented so that their main acoustic axis was vertically directed towards the floor of the tank and they were suspended at a depth of 0.12 m below the water surface. The two speakers were driven using a 2 channel Skytronic AV Digital Sound Amplifier. The playback system was controlled via a National Instruments 9264 analogue output card using MATLAB© running on a Laptop PC. An additional aquarium housed a sampling hydrophone to measure the instantaneous sound field below the sediment-water interface. The acoustic system was monitored throughout the experiment using a Bruel and Kjaer 8103 hydrophone, amplified through a Bruel and Kjaer type 2635 charge amplifier, running on a mains electrical supply and the data was captured via a National Instruments 9222 analogue input module. The monitoring comprised of recording 1 s averaged sound pressure levels, with 1 min spectra. The time series of the sound pressure levels and the spectra were stored at 1 min intervals. The data capture was controlled via MATLAB© running on a laptop PC. The acoustic system was calibrated using a Bruel and Kjaer 4229 piston phone. Prior to, and on completion of, the experimental phase the acoustic field emitted by the loudspeakers was measured. The transfer function (sound propagation) from the speakers to a grid of measurement points on a radial line from the centre of the tank was computed.

### Measures of tissue biochemistry

To provide data on the balance of metabolic process (see Supplementary Note 1) in the individuals exposed to each sound field, measures of tissue glucose and lactate were assayed. These data provide information on subtle, and potentially transitory, significant impacts to organism physiology and individual performance; mechanisms that ultimately underpin the contribution that individuals and, collectively, populations make to ecosystem processes. For *R. philippinarum*, samples of the adductor muscles were flash frozen in liquid nitrogen from 1 ind. aquarium^−1^ (n = 5). All tissues were stored at −80 °C prior to analysis. Each valve from every individual was stored at −20 °C to allow whole animal and flesh weight to be determined (Supplementary Table S2). For *N. norvegicus* the individual whole wet weight was recorded from 1 ind. aquarium^−1^ (n = 5) before flash freezing (Supplementary Table S2). Thereafter, tissue glucose and lactate were determined on tail muscle sample blocks, which were dissected from the frozen specimens. For *A. filiformis*, all individuals were flash frozen. For the glucose and lactate determination two brittlestars were pooled from each aquarium, wet weighed and processed together as whole animals (n = 5 per run) (Supplementary Table S2). All tissues were processed by first grinding in a pestle and mortar with liquid nitrogen. The powdered tissue was then deproteinized by the addition of five volumes of 0.6 M perchloric acid and homogenized before centrifugation at 4000 *g*, 5 °C for 10 minutes. Acid extract supernatants were neutralized with the stepwise addition of quantitative volumes of 2M potassium hydroxide and the precipitate was again removed by centrifugation at 4000 *g*, 5 °C for 10 minutes. The acid extracts of the ophiuroids were first were decolourized before being neutralized. 0.02 g of polyvinylpyrrolidone (PVPP) was added to 1 ml of the acid extract from each ophiuroid pair, briefly vortexed and then centrifuged at 5000 g for 10 minutes at 5 °C. Decolourized samples were then neutralized as described above. All neutralised extracts were stored at −80 °C before glucose and lactate assays were performed.

Glucose concentrations in the tissue extracts were determined using a hexokinase assay (Glucose (HK) Assay Kit, GAHK-20) according to the manufacturer’s protocol (Sigma Aldrich, Dorset, UK). Briefly, the glucose in the sample was converted to glucose-6-phophate in a reaction catalysed with hexokinase. The glucose-6-phosphate (G6P) was then oxidized to 6-phosphogluconate in the presence of oxidized nicotinamide adenine dinucleotide (NAD+), in a reaction catalyzed by glucose-6-phosphate dehydrogenase (G6PDH); a reaction which produced an equimolar amount of reduced NADH. The NADH produced was directly proportional to glucose concentration and was measured at 340 nm using a 1-cm light path cuvette. Sample glucose concentrations were converted to milligrams per gram wet flesh weight (mg gWW^−1^). Lactate concentrations in the tissue extracts were determined using a lactate dehydrogenase assay available as a kit (L-Lactic Acid kit, K-LATE 12/12) according to the manufacturer’s protocol (Megazyme International Limited, Co. Wicklow, Ireland).

### Measures of faunal behaviour

Sediment reworking (bioturbation) by benthic fauna was visualized non-invasively using a sediment profile imaging camera (f-SPI^63^) and fluorescent-dyed sediment particles (luminophores). Luminophores (125–250 μm: *R. philippinarum* and *A. filiformis*, 30 g aquarium^−1^; *N. norvegicus*, 120 g aquarium^−1^) were added and the average concentration of particles within the sediment profile was characterised from analysis of stitched images (n = 4, each side of the aquarium; *R. philippinarum, 6676 pixels*; *A. filiformis*, 7920 pixels; *N. norvegicus*, 14464 pixels) taken at the end of the incubation under ultra-violet light^64^. From these data, the median (^f-SPI^L_med_, typical short-term depth of mixing), maximum (^f-SPI^L_max_, maximum extent of mixing over the long-term) and mean (^f-SPI^L_mean_, time dependent indication of mixing) mixed depth of particle redistribution were calculated^65^. Surface boundary roughness, an indication of surficial reworking, was also determined (SBR, = range of sediment-water interface elevation). Bioirrigation activity was estimated from changes in water column concentrations of an inert tracer, (Sodium bromide, NaBr, dissolved in seawater [Br^−^] = 800 ppm, 5 mM, stirred into the overlying seawater) on day 6 of each experimental run. Water samples (5 ml) were taken at 0 and 4 h and immediately frozen (−18 °C). [Br^−^] was analysed using colorimetric analysis using a FIAstar 5000 flow injection analyzer (FOSS Tecator, Höganäs, Sweden). As bioirrigation activity reduces water column [Br^−^], negative values (∆ [Br^−^], mg L^−1^) indicate increased infaunal activity.

### Statistical analysis

Linear regression models were developed for the dependent variables (mean, median and maximum depth of particle redistribution, SBR, ∆ [Br^−^], and tissue glucose and lactate) with the independent nominal variable sound field (ambient, CBN, IBN). Where there was evidence of a violation of homogeneity of variance, the data were analysed using a *VarIdent* variance-covariate structure and a generalised least squares (GLS) estimation procedure to allow the residual spread to vary with individual explanatory variables^66^. We determined the optimal fixed-effects structure for each regression model using backward selection informed by Akaike Information Criteria (AIC) and inspection of model residual patterns. For the GLS analyses, the optimal variance covariate structure was determined using restricted maximum likelihood (REML) estimation; the initial regression model without variance structure is compared to the equivalent GLS model incorporating specific variance structures using AIC and visualisation of model residuals. The optimal fixed structure is then determined by applying backward selection using the likelihood ratio test obtained by maximum likelihood (ML) estimation. All analyses were performed in R^67^ and GLS analyses were conducted using the *nlme* package^68^.

## Additional Information

**How to cite this article**: Solan, M. *et al.* Anthropogenic sources of underwater sound can modify how sediment-dwelling invertebrates mediate ecosystem properties. *Sci. Rep.* **6**, 20540; doi: 10.1038/srep20540 (2016).

## Supplementary Material

Supplementary Information

Supplementary Information

Supplementary Information
