# Supplementary material for: Anthropogenic sources of underwater sound can modify how sediment-dwelling invertebrates mediate ecosystem properties
Source: Sci Rep. 2016 Feb 5;6:20540. doi: 10.1038/srep20540 (PMC4742813; doi:10.1038/srep20540)
Supplement: Supplementary Information [file srep20540-s1.pdf]

Supplementary Material

Anthropogenic sources of underwater sound can modify how sediment-dwelling invertebrates mediate ecosystem properties

Martin Solan[1], Chris Hauton[1], Jasmin A. Godbold[1,2], Christina L. Wood[1], Timothy G. Leighton[3] & Paul White[3]

[1]Ocean and Earth Science, National Oceanography Centre, Southampton, University of Southampton, Waterfront Campus, European Way, Southampton, SO14 3ZH. [2]Centre for Biological Sciences, Faculty of Natural and Environmental Sciences, University of Southampton, Highfield Campus, Southampton, SO17 1BJ. [3]Institute of Sound & Vibration Research, Faculty of Engineering and the Environment, University of Southampton, Southampton. SO17 1BJ.

Abbreviations: RP, *Ruditapes philippinarum*; NN, *Nephrops norvegica*; AF, *Amphiura filiformis*; Sound [Ambient, CBN, IBN], where: Ambient, ambient sound field; CBN, continuous broadband noise; and IBN, Impulsive broadband noise.

Figures: In all instances, data points (open circles, n = 5 per sound field treatment) have been horizontally jittered for clarity.

Supplementary Note 1

Concentrations of glucose within tissues represent the balance between glycogenolysis (i.e. the breakdown of energy stores of glycogen to form monomers of glucose for glycolysis) and glycogenesis (i.e. the conversion of excess glucose into glycogen for long term storage). Glucose is the starting substrate for the glycolysis in the metabolism of all metazoan cells.

In tissues that are functionally or environmentally hypoxic or anoxic the reduction in cellular oxygen prevents the generation of large amounts of ATP through oxidative phosphorylation within the mitochondria[1]. In these circumstances pyruvate accumulates within the cytoplasm of the cells through continued glycolysis. To remove that pyruvate, preventing its accumulation pushing the equilibrium of glycolysis towards glucose, the accumulated pyruvate is converted to lactate/lactic acid[2] via anaerobic metabolic pathways. The accumulation of lactic acid within tissues can, if unchecked, result in cellular acidosis that impacts the metabolic activity of cells and tissues. The accumulation of lactic acid in tissues has been widely used to infer perturbation to aerobic metabolism as well as the imposition of cellular and metabolic stress in marine invertebrates[3].

Hence, in organisms exposed to different sound fields, an increase in tissue glucose may indicate the increased catabolism of energy reserves as a requirement for increased glycolytic activity. Recorded increases in tissue lactate in exposed organisms may suggest compromised behavior(s) and reduced respiratory activity leading to a predominance of anaerobic metabolism to supply energetic requirements.

1. Schiedek, D. & Zebe, E. Functional and environmental anaerobiosis in the razor-clam Ensis directus (Mollusca: Bivalvia). *Marine Biology*. 94, 31-37 (1987).

2. Callow, P. & Townsend, C.R. Resource utilization in growth. In: Townsend C.R. & Callow, P. (eds.) *Physiological Ecology: an Evolutionary Approach to Resource Utilization*. Blackwell Scientific Publications, Oxford. (1981).

3. Albalat, A., Sinclair, S., Laurie, J., Taylor, A., Neil, D. Targeting the live market: Recovery of Norway lobsters *Nephrops norvegicus* (L.) from trawl-capture as assessed by stress-related parameters and nucleotide breakdown. J*ournal of Experimental Marine Biology and Ecology*. 395, 206–214 (2010).

Supplementary Figure S1 | Summary of tissue concentration of glucose (mg gWW^{-1}) observed for (a) adductor muscle of *Ruditapes philippinarum*, (b) tail muscle of *Nephrops norvegicus* and (c) whole organism of *Amphiura filiformis*. Data is shown for information only, as we found no effect of sound field.

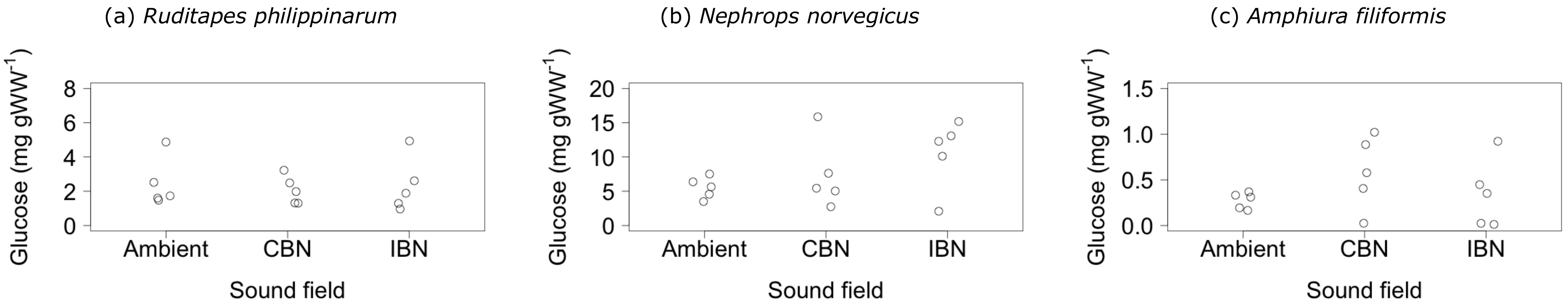

Supplementary Figure S2 | Summary of tissue concentration of lactate (mg gWW^{-1}) observed for (a) tissue of *Ruditapes philippinarum*, (b) abdominal muscle of *Nephrops norvegicus* and (c) whole organism of *Amphiura filiformis*. Data is shown for information only, as we found either no effect or, in the case of *R. philippinarum*, an insignificant but marginal effect (see Supplemental Model S1) of sound field.

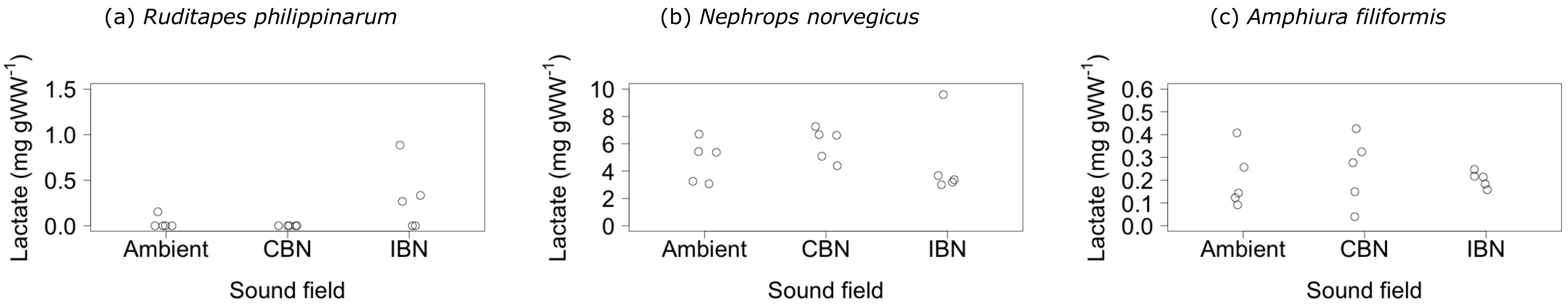

Supplementary Figure S3 | Summary of the Maximum mixed depth of particle reworking ($^{f\text{-}SPI}L_{max}$, cm) for (a) *Ruditapes philippinarum* and (b) *Amphiura filiformis*. Data is shown for information only, as we found no effect of sound field.

(a) *Ruditapes philippinarum* (b) *Amphiura filiformis*

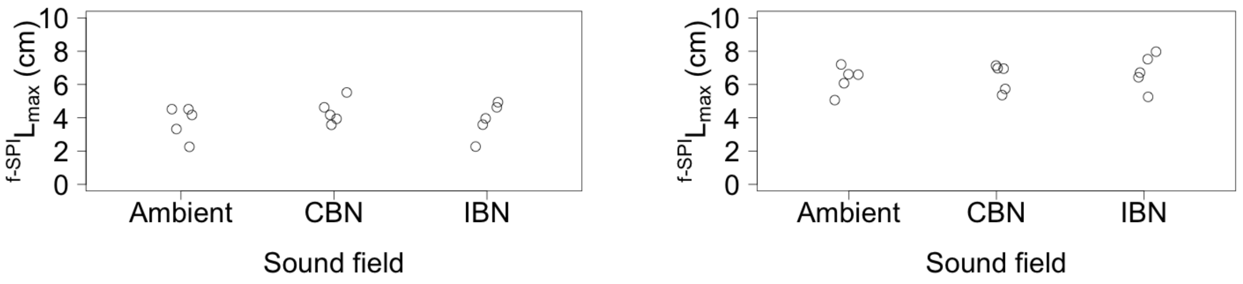

Supplementary Figure S4 | Summary of the Mean mixed depth of particle reworking ($^{f\text{-}SPI}L_{mean}$, cm) for (a) *Ruditapes philippinarum*, (b) *Nephrops norvegicus* and (c) *Amphiura filiformis*. Data is shown for information only, as we found no effect of sound field.

(a) *Ruditapes philippinarum* (b) *Nephrops norvegicus* (c) *Amphiura filiformis*

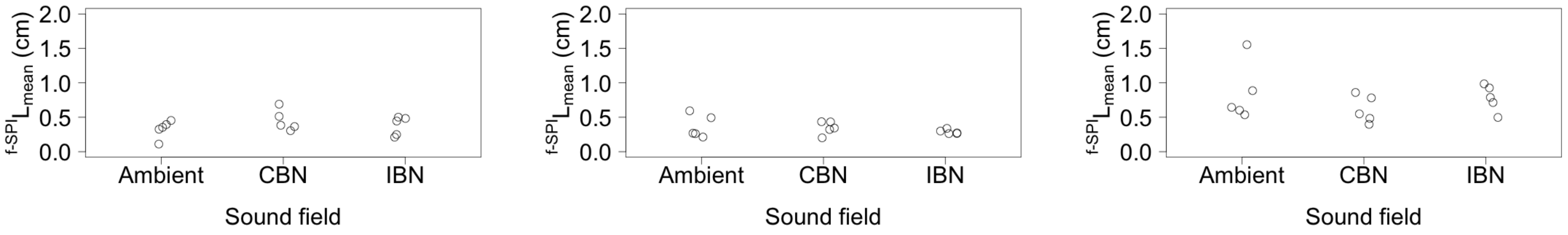

Supplementary Figure S5 | Summary of the Median mixed depth of particle reworking ($^{f\text{-}SPI}L_{med}$, cm) for (a) *Ruditapes philippinarum*, (b) *Nephrops norvegicus* and (c) *Amphiura filiformis*. Data is shown for information only, as we found no effect of sound field.

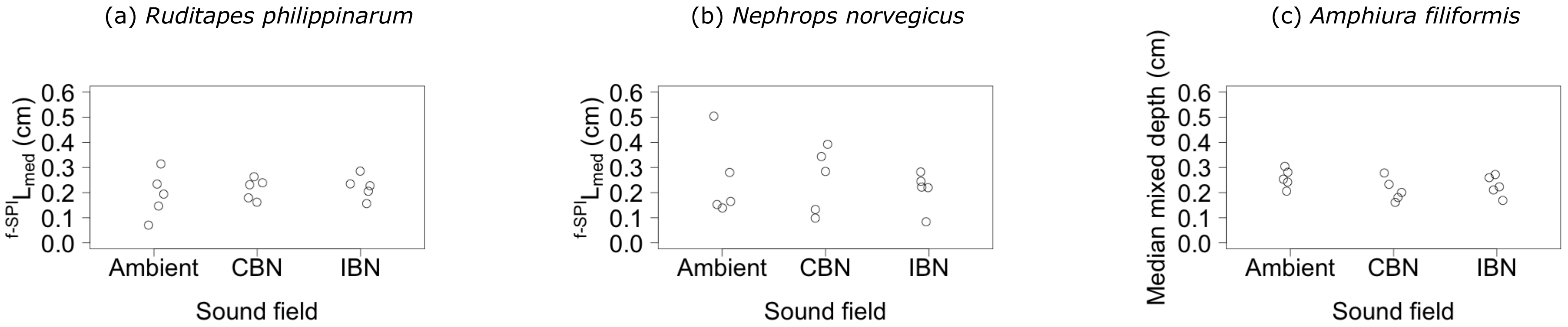

Supplementary Figure S6 | Summary of Surface Boundary Roughness (SBR, cm) observed for (a) *Nephrops norvegicus* and (b) *Amphiura filiformis*. Data is shown for information only, as we found no effect of sound field.

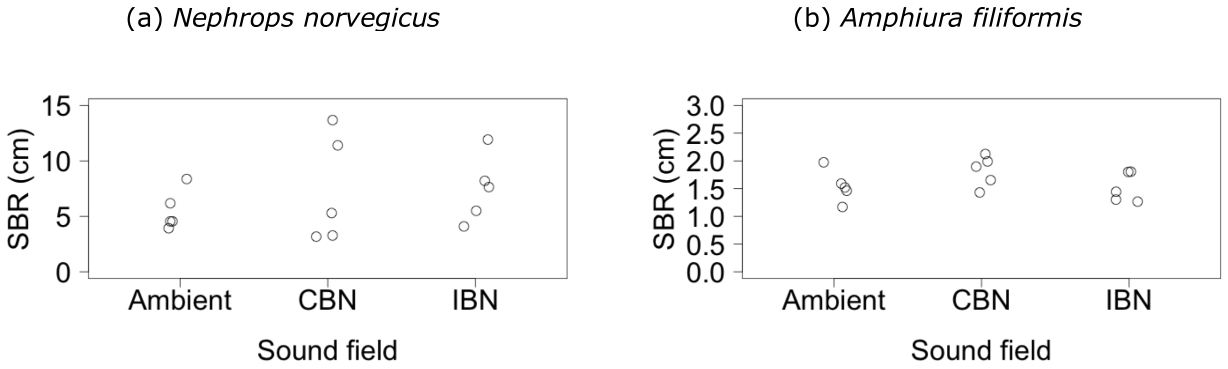

Supplementary Figure S7 | Summary of bioirrigation activity ($\Delta[Br^-]$, mg L^{-1}) observed for *Amphiura filiformis*. Data is shown for information only, as we found no effect of sound field. Negative values indicate increased bioirrigation activity.

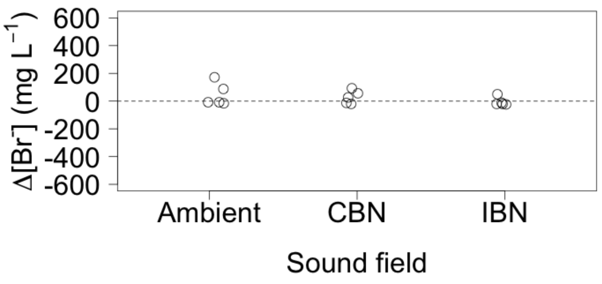

Statistical model summary

Summary of the statistical models. For each model, we list the initial linear regression model and, where applicable, the minimal adequate model with or without GLS estimation (including weightings that account for heterogeneity of variance), and a summary of the coefficient table. The coefficients indicate the relative performance of each treatment level relative to the re-leveled baseline (as indicated). Coefficients ± SE and t-values are presented alongside corresponding significance values (in parentheses) for each pairwise comparison.

Supplementary Model S1| Tissue concentrations of lactate (mg gWW^{-1}) for *Ruditapes philippinarum*.

Initial linear regression model:

lm(Lactate~ as.factor(Sound))

Minimal adequate model:

lm(4th root Lactate~ as.factor(Sound))

Analysis of Variance Table:

	Df	Sum Sq	Mean Sq	F value	Pr(>F)
as.factor(Sound)	2	0.64729	0.32364	3.3782	0.06858*
Residuals	12	1.14964	0.09580		

* marginal effect, see Supplementary Figure S2.

Supplementary Model S2 | Maximum mixed depth of particle reworking (${}^{f\text{-}SPI}L_{max}$, cm) for *Nephrops norvegicus*.

Initial linear regression model:

lm(${}^{f\text{-}SPI}L_{max}$ ~ as.factor(Sound))

Minimal adequate model:

gls(${}^{f\text{-}SPI}L_{max}$ ~ as.factor(Sound),weights = varIdent(form = ~1|as.factor(Sound)), method = 'REML')

Coefficient Table:

Intercept ± SE (when baseline is for an ambient sound field): 7.014216 ± 0.6580706, t = 10.658760, p <0.0001).

	Ambient	CBN	IBN
Ambient	-	-3.447493 ± 1.2804123<br>-2.692486<br>(0.0196)	-4.601804 ± 0.7107578<br>-6.474504<br>(0.0000)
CBN	3.447493 ± 1.280412<br>2.692486<br>(0.0196)	-	-1.154312 ± 1.130716<br>-1.020868<br>(0.3275)
IBN	4.601804 ± 0.7107578<br>6.474504<br>(0.0000)	1.154312 ± 1.1307160<br>1.020868<br>(0.3275)	-

Supplementary Model S3 | Surface boundary roughness (SBR, cm) for *Ruditapes philippinarum*.

Initial linear regression model:

lm(SBR ~ as.factor(Sound))

Minimal adequate model:

gls(SBR ~ as.factor(Sound),weights = varIdent(form = ~1|as.factor(Sound)), method = 'REML')

Coefficient Table:

Intercept ± SE (when baseline is for an ambient sound field): 1.7835587 ± 0.6443595, t = 7.685077, p <0.0001).

	Ambient	CBN	IBN
Ambient	-	-0.7915880 ± 0.2487526<br>-3.182230<br>(0.0079)	-0.3928419 ± 0.4496162<br>-0.873727<br>(0.3994)
CBN	0.7915880 ± 0.2487526<br>3.182230<br>(0.0079)	-	0.3987460 ± 0.3953601<br>1.008564<br>(0.3331)
IBN	0.3928419 ± 0.4496162<br>0.873727<br>(0.3994)	-0.3987460 ± 0.3953601<br>-1.008564<br>(0.3331)	-

Supplementary Model S4 | Bioirrigation ($\Delta[Br^-]$, mg L^{-1}) for *Ruditapes philippinarum*.

Initial linear regression model:

lm(Δ[Br⁻] ~ as.factor(Sound))

Minimal adequate model:

gls(Δ[Br⁻] ~ as.factor(Sound),
weights = varIdent(form = ~1|as.factor(Sound)), method = 'REML')

Coefficient Table:

Intercept ± SE (when baseline is for an ambient sound field): -330.6067 ± 62.53264, t = -5.286946, p =0.0002).

	Ambient	CBN	IBN
Ambient	-	779.1041 ± 64.39211<br>12.099372<br>(0.0000)	289.5820 ± 64.37899<br>4.498083<br>(0.0007)
CBN	-779.1041 ± 64.39211<br>-12.09937<br>(0.0000)	-	-489.5220 ± 21.68721<br>-22.57192<br>(0.0000)
IBN	-289.5820 ± 64.37899<br>-4.498083<br>(0.0007)	489.5220 ± 21.68721<br>22.57192<br>(0.0000)	-

Supplementary Model S5 | Bioirrigation ($\Delta[Br^-]$, mg L^{-1}) for *Nephrops norvegicus*.

Initial linear regression model:

lm(Δ[Br⁻] ~ as.factor(Sound))

Minimal adequate model:

gls(Δ[Br⁻] ~ as.factor(Sound),
weights = varIdent(form = ~1|as.factor(Sound)), method = 'REML')

Coefficient Table:

Intercept ± SE (when baseline is for an ambient sound field): 142.77061 ± 37.94334, t = 3.762732, p =0.0027).

	Ambient	CBN	IBN
Ambient	-	-151.22820 ± 38.10035<br>-3.969208<br>(0.0019)	-38.61867 ± 79.40675<br>-0.486340<br>(0.6355)
CBN	151.22820 ± 38.10035<br>3.969208<br>(0.0019)	-	112.60953 ± 69.84035<br>1.612385<br>(0.1329)
IBN	38.61867 ± 79.40675<br>0.486340<br>(0.6355)	-112.60953 ± 69.84035<br>-1.612385<br>(0.1329)	-

Supplementary Table S1 | The standard deviations (SD, n = 5) for all response variables (lactate, glucose, $^{f\text{-}SPI}L_{max}$, $^{f\text{-}SPI}L_{mean}$, $^{f\text{-}SPI}L_{med}$, SBR and $\Delta[Br^-]$) for (a) *Ruditapes philippinarum*, (b) *Nephrops norvegicus*, and (c) *Amphiura filiformis*. Comparisons of the standard deviation under sound exposure treatments (CBN and/or IBN) relative to the ambient treatment are indicated.

	Sound exposure treatment			Pairwise comparisons			
Response variable	Ambient	CBN	IBN	CBN > Ambient?	IBN > Ambient?	CBN or IBN> Ambient?	IBN > CBN?
(a) *Ruditapes philippinarum*							
Lactate	0.069	0	0.362	No	Yes	Yes	Yes
Glucose	1.419	0.818	1.580	No	Yes	Yes	Yes
$\Delta[Br^-]$	139.827	34.352	34.229	No	No	No	No
$^{f\text{-}SPI}L_{max}$	0.970	0.750	1.044	No	Yes	Yes	Yes
$^{f\text{-}SPI}L_{mean}$	0.130	0.154	0.137	Yes	Yes	Yes	No
$^{f\text{-}SPI}L_{med}$	0.091	0.043	0.047	No	No	No	Yes
SBR	0.519	0.200	0.861	No	Yes	Yes	No
(b) *Nephrops norvegicus*							
Lactate	1.558	1.205	2.821	No	Yes	Yes	Yes
Glucose	1.557	5.071	5.066	Yes	Yes	Yes	No
$\Delta[Br^-]$	84.844	7.726	155.977	No	Yes	Yes	Yes
$^{f\text{-}SPI}L_{max}$	1.471	2.456	0.600	Yes	No	Yes	No
$^{f\text{-}SPI}L_{mean}$	0.167	0.096	0.032	No	No	No	No
$^{f\text{-}SPI}L_{med}$	0.154	0.129	0.075	No	No	No	No
SBR	1.801	4.866	2.987	Yes	Yes	Yes	No
(c) *Amphiura filiformis*							
Lactate	0.129	0.151	0.034	Yes	No	Yes	No
Glucose	0.089	0.395	0.373	Yes	Yes	Yes	No
$\Delta[Br^-]$	82.677	47.423	31.077	No	No	No	No
$^{f\text{-}SPI}L_{max}$	0.804	0.821	1.051	Yes	Yes	Yes	Yes
$^{f\text{-}SPI}L_{mean}$	0.418	0.198	0.192	No	No	No	No
$^{f\text{-}SPI}L_{med}$	0.038	0.046	0.041	Yes	Yes	Yes	No
SBR	0.289	0.277	0.264	No	No	No	No

Supplementary Table S2 | Whole wet weight‡ of individuals used for the assay of metabolites

Species	Individual	Whole wet weight (g)		
		Control	CBN	IBN
Ruditapes philippinarum†	1	40.72	32.20	30.13
	2	25.91	29.60	38.60
	3	42.10	26.59	25.86
	4	31.41	57.77	21.22
	5	24.32	26.02	23.72
Nephrops norvegicus	1	48.36	63.18	56.92
	2	87.91	49.96	62.66
	3	91.31	68.43	61.70
	4	65.14	48.33	55.71
	5	65.19	72.59	68.56
*Amphiura filiformis**	1	1.89	1.99	1.88
	2	1.47	1.81	1.78
	3	1.52	1.84	1.53
	4	2.03	2.16	2.04
	5	1.77	2.00	1.75

‡ wet weights available only, as the tissues were homogenised in liquid nitrogen immediately after weighing.

† for *Ruditapes philippinarum* weight includes shell and tissue.

* for *Amphiura filiformis* two individuals were pooled before homogenisation in liquid nitrogen.

Sound fields

Supplementary sound field recordings | Sound field files used in this study are available online in MPEG 3 (Moving Picture Experts Group) format for continuous broadband noise (**Supplementary Sound 1**) and impulsive broadband noise (**Supplementary Sound 2**).

Supplementary Figure S8 | Power Spectral Density (PSD) of the CBN source (shipping noise). This is computed using 1 Hz resolution using a Hanning window. The levels shown represent those recorded in the tank during replay.

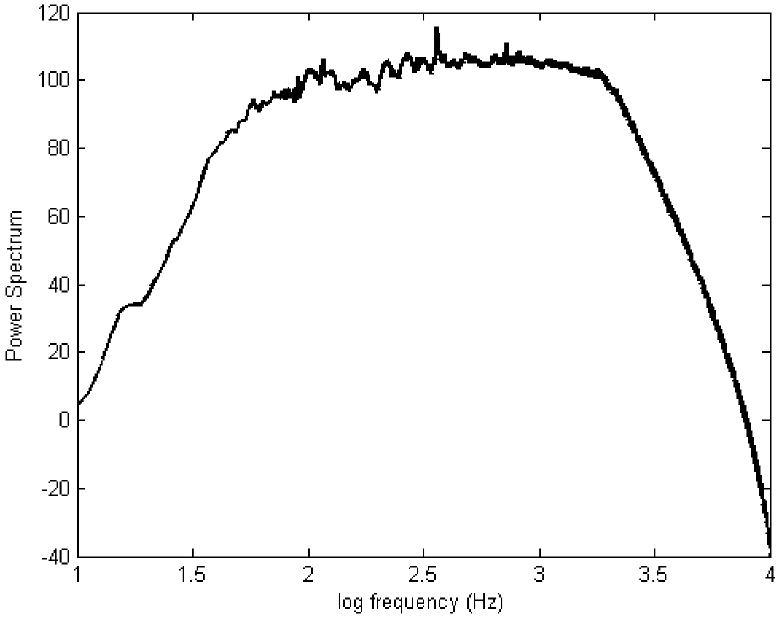

Supplementary Figure S9 | Power Spectral Density (PSD) of the IBN source (pile driving). This is evaluated by computing the spectrum of a 1 second window centered on the each of the 85 impulses in the recording and the overall PSD is the average of those spectra. The levels shown represent those recorded in the tank during replay.

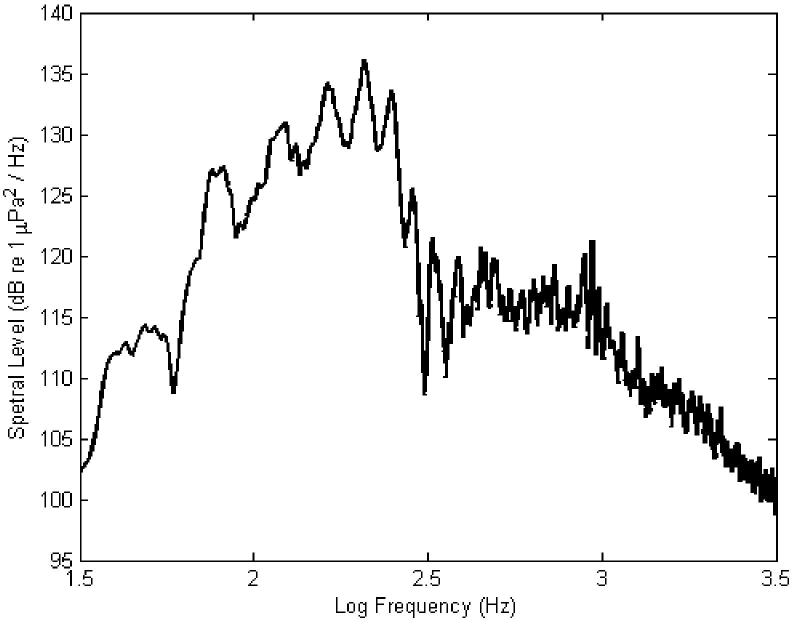

ENDS.
